# A Glucose-Utilizing Strain, *Cupriavidus euthrophus* B-10646: Growth Kinetics, Characterization and Synthesis of Multicomponent PHAs

**DOI:** 10.1371/journal.pone.0087551

**Published:** 2014-02-24

**Authors:** Tatiana Volova, Evgeniy Kiselev, Olga Vinogradova, Elena Nikolaeva, Anton Chistyakov, Aleksey Sukovatiy, Ekaterina Shishatskaya

**Affiliations:** 1 Institute of Biophysics of Siberian Branch of Russian Academy of Sciences, Krasnoyarsk, Russian Federation; 2 Siberian Federal University, Krasnoyarsk, Russian Federation; 3 Shemyakin-Ovchinnikov Institute of Bioorganic Chemistry of the Russian Academy of Science, Moscow, Russian Federation; University Paris South, France

## Abstract

This study investigates kinetic and production parameters of a glucose-utilizing bacterial strain, *C. eutrophus* B-10646, and its ability to synthesize PHA terpolymers. Optimization of a number of parameters of bacterial culture (cell concentration in the inoculum, physiological activity of the inoculum, determined by the initial intracellular polymer content, and glucose concentration in the culture medium during cultivation) provided cell concentrations and PHA yields reaching 110 g/L and 80%, respectively, under two-stage batch culture conditions. Addition of precursor substrates (valerate, hexanoate, propionate, γ-butyrolactone) to the culture medium enabled synthesis of PHA terpolymers, P(3HB/3HV/4HB) and P(3HB/3HV/3HHx), with different composition and different molar fractions of 3HB, 3HV, 4HB, and 3HHx. Different types of PHA terpolymers synthesized by *C. eutrophus* B-10646 were used to prepare films, whose physicochemical and physical-mechanical properties were investigated. The properties of PHA terpolymers were significantly different from those of the P3HB homopolymer: they had much lower degrees of crystallinity and lower melting points and thermal decomposition temperatures, with the difference between these temperatures remaining practically unchanged. Films prepared from all PHA terpolymers had higher mechanical strength and elasticity than P3HB films. In spite of dissimilar surface structures, all films prepared from PHA terpolymers facilitated attachment and proliferation of mouse fibroblast NIH 3T3 cells more effectively than polystyrene and the highly crystalline P3HB.

## Introduction

Global production and consumption of chemical compounds derived from nonrenewable natural resources increase rapidly, leading to accumulation of non-recyclable wastes and, thus, coming into conflict with environmental protection activities [1]. Annual production of synthetic plastics approaches 300 million t., and they largely accumulate in landfills. Large areas of land, including agricultural fields, are occupied by new landfills; polymer garbage occludes sewers and drain lines in the cities and pollutes water ecosystems, impairing water quality and endangering the biota of the Global Ocean [2], [3]. One way to reduce human impact on ecosystems is to replace synthetic polymers by new materials that are biodegraded in nature into components harmless to the environment, which can be involved in global material cycles [4].

At the present time, the most widely used and actively developed biodegradable biomaterials are synthetic and natural polymers such as aliphatic polyesters, polyamides, segmental polyester urethanes, and complex polyesters of aliphatic hydroxy carboxylic acids with different structures. Among the most promising materials are biodegradable polyesters of monocarboxylic acids, mainly represented by polylactides and polyglycolactides. The second most promising and best-studied biodegradable materials are polymers of microbial origin – polyhydroxyalkanoates (PHAs). These polymers are synthesized by prokaryotic microorganisms, which usually accumulate them under unbalanced growth conditions as an endogenous carbon and energy source; the basic properties of PHAs are similar to those of polypropylene. Potential applications of PHAs are very wide: they can be used in agriculture, municipal engineering, radio electronics, medicine, and pharmacology [5]–[10].

Large corporations and companies such as Monsanto, Metabolix Inc., Tepha, Proctor & Gamble, Berlin Packaging Corp., and Merk are engaged in commercial production of PHAs trademarked as Biopol®, TephaFLEX™, DegraPol/btc®, Nodax™, Mirel™, etc. [8], [11]. The high cost of PHAs is an obstacle to their large-scale production, but it may be reduced by using more productive microorganisms and low-cost feedstocks for PHA synthesis.

Among promising PHA producers are representatives of the genus *Cupriavidus* (formerly known as *Wautersia, Ralstonia, Alcaligenes, Hydrogenomonas*) [12], which have high growth rates and are able to synthesize PHAs from various substrates [13]–[15]. *Cupriavidus* species include recombinant and wild-type strains that are capable of synthesizing not only poly-3-hydroxybutyrate, a high-crystallinity and brittle polymer, but also PHA copolymers containing monomer units with carbon chains of different lengths (hydroxybutyrate, hydroxyvalerate, hydroxyhexanoate, etc.) [16]–[21].

The ability to synthesize PHAs with different chemical structures is a particularly useful property, providing the basis for the production of tailor-made materials [21]. Recently, researchers have shown an increased interest in cultivation of bacteria that are able to produce PHA bi- and ter-polymers with widely varying properties (melting temperature, crystallinity, mechanical strength) [5], [7], [22] and enhanced biocompatibility [23]–[25]. Synthesis of PHA copolymers is usually achieved, however, by supplementing the culture medium with different additional precursor carbon sources (valerate, hexanoate, propionate, butyrolactone, etc.), which are toxic to bacteria and, thus, impair the total productivity of biosynthesis and reduce polymer yields [20], [26], [27]. Therefore, it is important to find and/or engineer strains that would be tolerant to these compounds.

It is also important to reduce the cost of the substrates for PHA synthesis. The currently used substrates include both individual compounds (sugars, organic acids, alcohols, C_1_ compounds, etc.) and different wastes [20]. *Cupriavidus* species have a broad organotrophic potential and are able to synthesize PHAs from mixtures of CO_2_ and H_2_ and from organic substrates, but fructose is the only sugar that they can utilize. However, as these bacteria are mutable organisms, glucose-utilizing strains can be engineered and then cultivated on glucose, which is a lower-cost substrate.

In a previous study we engineered a mutant glucose-utilizing strain, *Ralstonia eutropha* B8562, capable of synthesizing PHA copolymers containing poly-3-hydroxybutyrate (P3HB) and minor fractions of 3-hydroxyvalerate (3HV) [28]. The strain, however, showed high sensitivity to glucose, which inhibited its growth at concentrations above 15 g/L, and we failed to achieve high yields.

The recently isolated glucose-utilizing strain, *Cupriavidus eutrophus* B-10646, is glucose tolerant. Moreover, it shows an enhanced tolerance to a number of organic precursor substrates necessary for the synthesis of PHAs with different chemical structures. This strain is capable of synthesizing copolymers of 3-hydroxybutyrate (3HB) and 4-hydroxybutyrate (4HB) [29] and copolymers of 3HB, 3HV and/or 3-hydroxyhexanoate (3HHx) [30].

The purpose of this study was to optimize conditions of cultivation of *C. eutrophus* B-10646 cells synthesizing polyhydroxyalkanoates, using glucose as the main growth substrate, and investigate physicochemical, mechanical, and biological properties of PHA terpolymers.

## Materials and methods

### Materials

**Bacterial strain:** The strain used in this study was *C. eutrophus* B-10646, registered in the Russian Collection of Industrial Producers (RCIP). The strain has the following genetic traits: The GC-content in the DNA is 66%. Nucleotide sequences of the 16S rRNA gene of the B-10646 strain (1381 np) have been cloned and characterized; they are deposited into the GenBank database No. JQ695938. The strain is an obligate aerobe; a facultative chemolithoautotroph. It is oxidase positive. The strain has no hydrolytic enzymes. It is not a gelatin-liquefying or starch-hydrolyzing strain. It has a broad organotrophic potential and can use as carbon sources the following substances: sugars (glucose and fructose), amino acids (alanine, serine, leucine, histidine, tryptophan, glutamic acid, aspartic acid, and lysine), organic acids (oxalic, citric, succinic, fumaric, acetic, β- and γ-butyric, pentanoic, hexanoic, octanoic, and nonanoic acids), alcohols (ethanol and glycerol), γ-butyrolactone, CO_2_, and CO. As a nitrogen source, the strain utilizes nitrates, ammonium salts, urea, and amino acids. It is capable of synthesizing PHA copolymers containing short- and medium-chain-length monomer units.

**Media:** Schlegel’s mineral medium was used as a basic solution for growing cells [31]: Na_2_HPO_4_⋅H_2_O – 9.1; KH_2_PO_4_ – 1.5; MgSO_4_⋅H_2_O – 0.2; Fe_3_C_6_H_5_O_7_⋅7H_2_O – 0.025; CO(NH_2_)_2_ - 1.0 (g/L). The main carbon substrate was glucose, which was sterilized by membrane filtration using Opticap XL300 Millipore Express SHC filters (U.S.), in order to prevent the pH from falling. Nitrogen was provided in the form of urea, and, thus, no pH adjustment was needed. The pH level of the culture medium was stabilized at 7.0±0.1. A solution of iron citrate (5 g/L), which was used as a source of iron, was added to reach a concentration of 5 ml/L. Hoagland’s trace element solution was used: 3 ml of standard solution per 1 L of the medium. The standard solution contains H_3_BO_3_ – 0.288; CoCl_2_⋅6H_2_O – 0.030; CuSO_4_⋅5H_2_O – 0.08; MnCl_2_⋅4H_2_O – 0.008; ZnSO_4_⋅7H_2_O – 0.176; NaMoO_4_⋅2H_2_O – 0.050; NiCl_2_ – 0.008 (g/L).

Substrate feeding strategies were varied depending on the technique of bacterial cultivation employed: culture in flasks in a shaker or culture in a fermentation system.

**Growth conditions:** Cells were grown in batch culture, as developed previously for PHA synthesis [17]. A two-stage process was used. In the first stage, cells were grown under nitrogen deficiency: the amount of nitrogen supplied in this stage was 60 mg/g cell biomass synthesized (i.e. 50% of the cell’s physiological requirements – 120 mg/g); the cells were cultured in complete mineral medium and with glucose flux regulated in accordance with the requirements of the cells. In the second stage, cells were cultured in nitrogen-free medium; the other parameters were the same as in the first stage. The temperature of the culture medium was 30±0.5°C and pH was 7.0±0.1.

Inoculum was produced using an Innova® 44 constant temperature incubator shaker (“New Brunswick Scientific”, U.S.). Inoculum was prepared by resuspending the museum culture maintained on agar medium. Museum culture was grown in 1- to 2-L glass flasks half-filled with saline liquid medium, with the initial concentration of glucose from 10 to 30 g/L. Depending on the length of this phase (10 to 40 h), cell concentration of the inoculum varied from 1 to 8 g/L and intracellular polymer content from 10 to 40% or more. In the phase of bacterial culture in the fermentor, the lowest initial cell concentration was 1 g per L.

Dynamics of accumulation of cell biomass and PHA by strain B-10646 was studied in a BioFlo-115 automated laboratory fermentor, with a 12-L fermentation vessel and the working volume of the culture from 3 L to 8 L, under strictly aseptic conditions. The fermentor is equipped with two turbine-type impellers: *d_i_*  =  0.075 m, the width of the impeller blade 0.030 m, the number of baffles in the fermentor 4, diameter and number of sparger holes 0.001 m and 10, respectively, jacket surface area 0.2 m^2^. The mass flow of the fermentor is controlled by the air flow rate and agitation speed; the latter can be varied from 300 to 1000 rpm, and, thus, the oxygen transfer rate, KLa, varies from 120 to 480 1/h. The fermentor is equipped with a control station with a liquid crystal display, which records the data of cultivation process, pH probes, O_2_ probes, a system for automatic substrate feeding, and a thermal stabilization system. The air was continuously pumped through the culture medium, using microbiological filters and an EL-200 air pump, of productivity 9 m^3^/h and pressure 19.6 kPa. The air is pumped into the culture medium automatically, at 1.5–3 L/min. Oxygen saturation level was maintained at 25-30%; as cell concentration increased, oxygen concentration began to decrease, and agitation speed increased (cascaded control).

When cell concentration reached 2.0–2.5 g/L, nitrogen (60 mg/g biomass) and glucose solution were fed to the culture medium in two separate flows. Substrate feed rates were regulated with a peristaltic pump. Nitrogen concentration in the culture medium was maintained at trace levels, and glucose concentration did not exceed 10 g/L. Nitrogen supply was stopped after 20–25 h, and the second phase was started; it lasted for 20–55 h, until P3HB reached (83±2)%.

Synthesis of PHA copolymers was achieved as follows: after 4–6 h of cultivation, nitrogen supply was discontinued, and the culture medium was supplemented with precursor substrates (γ-butyrolactone; propionic, valeric, and hexanoic acids in the form of potassium salts). Precursor substrates may be added in 1 to 3 portions in the first half of the first stage of the culture. Concentrations of these substrates in the culture medium were at levels close to the maximum tolerable concentrations for the given strain.

### Methods

**Monitoring process parameters:** During the course of cultivation, samples of culture medium were taken for analysis every 4 h: cell concentration in the culture medium was determined based on the weight of the cell samples dried at 105°C for 24 h (DCW) per 1 L. Cell concentration in the culture medium was monitored every hour by converting the optical absorbance at 440 nm of culture broth to dry cell weight by using a standard curve prepared previously.

Glucose concentration was determined using the “Glucose – FKD” kit, which contained chromogenic enzyme substrate and a calibrator (a glucose solution of a known concentration). Optical density of the study sample and calibration sample were compared photometrically with the optical density of the blank, with optical path length 10 mm at wavelength 490 nm. Glucose concentration in the samples was calculated using the following formula:
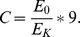



Concentrations of precursor substrates in the culture medium were controlled using chromatographic analysis of the culture medium samples, which was done after preliminary extraction with chloroform from acidified samples. Nitrogen concentration in the culture medium was analyzed at different time points, using a photometric method, with Nessler’s reagent. To measure concentrations of major and trace elements (S, K, Mg, P, Na, Ca, Fe, Cu, Mn, Mo, Zn, Co, Cr, Se), samples of the culture medium were taken periodically and measured using inductively coupled plasma atomic emission spectroscopy in an ICAP – 6000 Thermo system (Thermo Electron Corporation, U.S.).

PHA biosynthesis was evaluated based on cell concentration, polymer yield, the amount of the main growth substrate used, and process duration and productivity. Conventional methods were used to determine kinetic and production parameters of the culture. The cell biomass yield (X, kg/m^3^), the gas flow rate (Gr, kg/h), the yield coefficient of the culture (Y, kg biomass/kg), and the specific growth rate (µ, h^−1^) were calculated.

Specific growth rate of the culture (*µ*, h^−1^) was determined using the following equation: 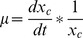



where *x_c_* is catalytic biomass, g/L;


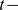
duration of cultivation**,** h.
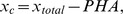



where 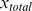 is total biomass

Specific rate of polymer synthesis (*µ_β_* **,** h^−1^) was determined using the following formula:
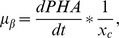



where *PHA* are initial and final intracellular polymer concentrations, g/L.

The yield coefficient of the culture from the substrate, *Y*, g/g, was calculated using the following formula: 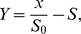



where *S* and *S*_0_ are the final and initial substrate concentrations.

Bacterial respiratory activity (*q*_min_) was measured polarographically, using a platinum electrode in a 2-cm^3^ container filled with incubation medium: the microbial culture sample was preliminarily incubated to achieve a stable level of endogenous respiration of cells; a carbohydrate substrate was added once into the incubation medium; dynamics of oxygen loss from the incubation medium was analyzed quantitatively. Oxygen consumption rate was expressed as mole/g·h.

**Analysis of PHA structure and physicochemical properties:** Intracellular PHA content at different time points was determined by analyzing samples of dry cell biomass. Intracellular PHA content and composition of extracted polymer samples were analyzed by a GC-MS (6890/5975C, “Agilent Technologies”, U.S.). Both lyophilized cells and extracted PHAs were subjected to methanolysis in the presence of sulfuric acid, and PHA was extracted and methyl esterified at 100°C for 4 h. Benzoic acid was used as an internal standard to determine total intracellular PHA [32], [33]. Monomer units were identified in the extracted and purified PHA samples based on their retention times and mass spectra.

^1^H NMR spectra of copolymer were recorded at room temperature in CDCl_3_ on a BRUKER AVANCE III 600 spectrometer (Germany) operating at 600.13 MHz.

Molecular weight and molecular-weight distribution of PHAs were examined using a gel permeation chromatograph (“Agilent Technologies” 1260 Infinity, U.S.) with a refractive index detector, using an Agilent PLgel Mixed-C column. Chloroform was the eluent, at a flow rate of 1.0 ml/min at 40°C. Typical sample volumes were 50 µl at a polymer concentration of 2 mg/ml. Narrow polydispersity polystyrene standards (Agilent, U.S.) were used to generate a universal calibration curve, from which molecular weights (weight average, M_w_, and number average, M_n_) and polydispersity (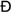) were determined. The measurement accuracy was 2%.

Thermal analysis of PHA specimens was performed using a DSC-1 differential scanning calorimeter (METTLER TOLEDO, Switzerland). Powdered samples (4.0 ±0.2 mg each) were placed into the aluminum crucible and compressed prior to measurement. Every sample was measured at least 3 times. Samples were preheated to 60°C and cooled to 25°C. The specimens were heated to temperatures from 25°C to 300°C, at 5°C×min-1 (measurement precision 1.5°C); melting point (Tm) and thermal decomposition temperature (Td) were determined from exothermal peaks in thermograms. The thermograms were analyzed using the STARe v11.0 software.

In order to determine the crystallinity of the PHAs, 3 film samples 2 cm in diameter and 0.15 mm thick were prepared from a 2% polymer solution in chloroform. The samples had a circular shape because during measurement the sample spins in a direction perpendicular to the surface. X-Ray structure analysis and determination of crystallinity of PHAs were performed employing a D8ADVANCE X-Ray powder diffractometer equipped with a VANTEC fast linear detector, using CuKa radiation (“Bruker, AXS”, Germany). The scan step was 0.016°, measurement time in each step 114 s, and scanning range from 5° to 60° (from 48° to 60° there only was a uniformly decreasing background); the registered parameter was intensity of X-rays scattered by the sample; 55°/0.016° = 3438 times. The degree of crystallinity was calculated as a ratio of the total area of crystalline peaks to the total area of the radiograph (the crystalline + amorphous components). Measurement accuracy: point measurement accuracy ± 0.4 PPS, with the lowest intensity 1.5 PPS and the highest intensity 32 PPS; the error in determination of the degree of crystallinity, which was calculated based on multiple measurements, was 2% or less.

**Analysis of PHA physical-mechanical properties:** The microstructure of the surface of PHA films was analyzed using scanning electron microscopy (TM 3000, Hitachi, Japan). Surface properties such as surface free energy (γ_S_), interfacial free energy (γ_SL_) and cohesive forces (*W*_SL_) (erg/cm^2^) were calculated based on the measured water contact angles (θ, degrees), using the de Gennes equations [34].

The roughness of film surface was determined using atomic-force microscopy (AFM) in semicontact mode (Smart SPM™, AIST-NT, Zelenograd, Russia). Sites of areas 20×20 µm were examined, and 2×2 µm sites with local maxima and minima were selected; they were micrographed at a higher resolution, and the roughness of each sample was calculated for three sites. Average roughness (Ra) and root mean squared roughness (Rq) were calculated based on 10 points, as the arithmetic averages of the absolute values of the vertical deviations of the five highest peaks and lowest valleys from the mean line of the profile of the 2×2 µm surface, using conventional equations [35].

Physical-mechanical properties of films prepared from PHAs with different compositions were investigated using an Instron 5565 electromechanical tensile testing machine (UK). Dumbbell-shaped samples 50 mm long, 6.1 mm wide, and 25–30 µm thick were prepared for studying physical-mechanical properties of the films. The thickness of films was measured prior to testing, using a “LEGIONER EDM-25-0.001” electronic digital micrometer.

Samples were maintained under normal conditions for at least two weeks to reach equilibrium crystallization. At least five samples were tested for each type of films. Measurements were conducted at room temperature; the clamping length of the samples was 30 mm. The speed of the crosshead was 3 mm/min at room temperature. Young’s modulus (E, MPa), tensile strength (σ, MPa) and elongation at break (ε, %) were automatically calculated by the Instron software (Bluehill 2, Elancourt, France). To obtain Young’s modulus, the software calculated the slope of each stress-strain curve in its elastic deformation region. Measurement error did not exceed 10%.

**Assays of cell attachment and viability:** Films were cut into disks of 10 mm diameter, using a mold cutter. The samples were packed using an NS 1000 shrink-wrapping machine (Hawo Gmbh, Germany) and sterilized with H_2_O_2_ plasma in the Sterrad NX system (Johnson & Johnson, U.S.) for 45 min.

**Assays of cell attachment and cell viability:** The ability of ultrafine PHA films to facilitate cell attachment was studied using NIH 3T3 mouse fibroblast cells. Cells were seeded into 24-well cell culture plates (Greiner Bio-One, U.S.) (1×10^3^ cells/ml per well). Cells were cultured in DMEM medium supplemented with 10% fetal bovine serum and a solution of antibiotics (streptomycin 100 µg/ml, penicillin 100 IU/ml) (Sigma) in a CO_2_ incubator with CO_2_ level maintained at 5%, at a temperature of 37°C. The medium was replaced every three days. Morphology of cells attached to the film surface was determined using DAPI and FITC fluorescent dyes (DNA and cytoplasm markers).

Cell viability was evaluated using MTT assay at Day 7 after cell seeding onto films. Reagents were purchased from Sigma–Aldrich. A 5% MTT solution (50 µl) and complete nutrient medium (950 µl) were added to each well of the culture plate. After 3.5 h incubation, the medium and MTT were replaced by DMSO to dissolve MTT-formazan crystals. After 30 min, the supernatant was transferred to the 96-well plate, and optical density of the samples was measured at wavelength 540 nm, using a Bio-Rad 680 microplate reader (Bio-Rad LABORATORIES Inc., U.S.). Measurements were performed in triplicate. The number of viable cells was determined from the calibration graph.

### Statistics

Statistical analysis of the results was performed by conventional methods, using the standard software package of Microsoft Excel. Arithmetic means and standard deviations were found. The statistical significance of results was determined using Student’s test (significance level: P ≤ 0.05).

## Results

### Growth kinetics of C. eutrophus B-10646 culture and PHA synthesis

In order to achieve efficient PHA synthesis by *C. eutrophus* B*-*10646 cells, experiments were performed to determine the limits of physiological effect of glucose on the cells and find out whether changes in the physiological activity of the inoculum and initial cell concentration of the inoculum could enhance process efficiency.

The experiments showed that for the study strain, the range of tolerable glucose concentrations in the culture medium was 5 to 35 g/L. Under conditions of unlimited glucose supply, specific growth rate of the cells reached 0.28 h^−1^; kinetic constants for this substrate, K_s_ and K_i_, found using conventional methods (the Monod model for limited growth and the Monod-Ierusalimsky model for inhibited growth) and the Lineweaver–Burk plots, were 0.011 and 0.12 mol/L, respectively. Glucose concentrations outside this range adversely affected cell yields. Thus, during fermentation, glucose should be added to the culture medium portion-wise or continuously, with a peristaltic pump, and its concentration should be carefully monitored.

**Figure 1:**
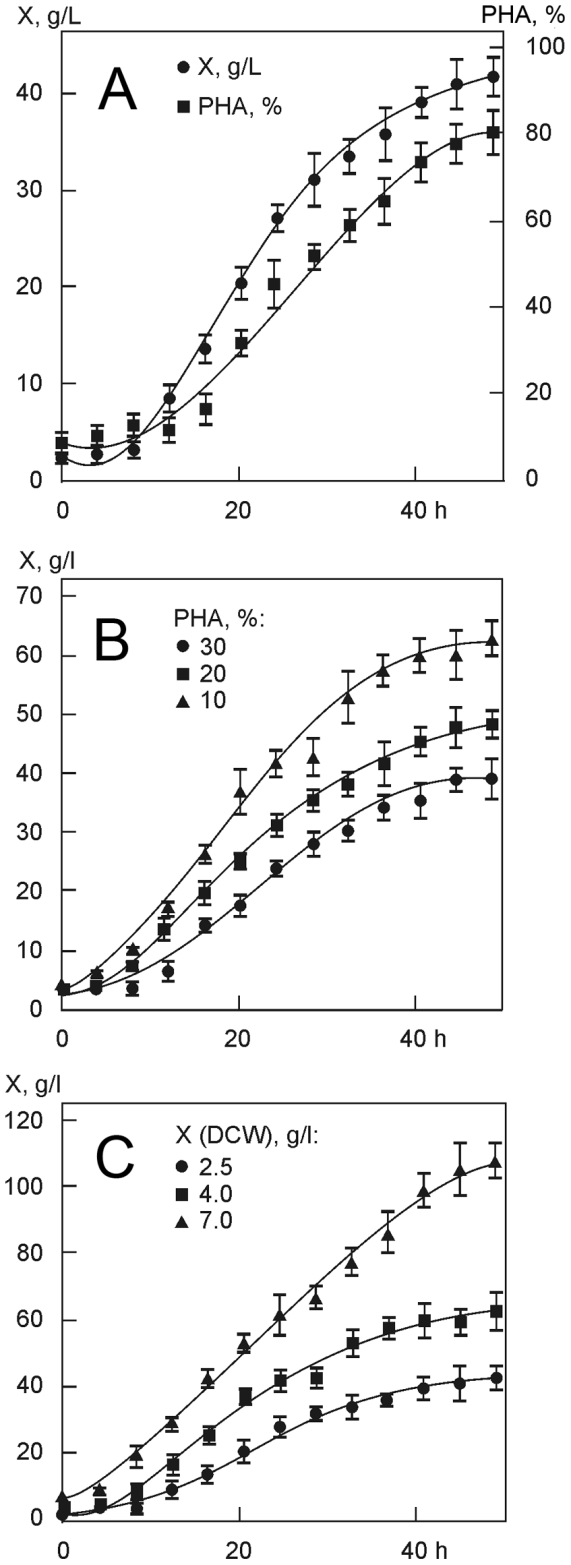
*C. eutrophus* B-10646 culture parameters under standard conditions of PHA synthesis (A); at varied initial polymer concentrations in the inoculum (B); and at varied cell concentrations in the inoculum (C).

Analysis of conditions influencing PHA accumulation by *C. eutrophus* B-10646 cells was performed in the experiment with a two-stage batch culture; in the first stage, cells were grown under nitrogen limitation and in the second – in nitrogen-free medium. A typical curve of cell yield and polymer accumulation in the cells grown on glucose, with the initial cell concentration in the inoculum (2.4±0.2) g/L, is shown in Figure 1A. In the first stage (20–25 h), under nitrogen deficiency (50% of the cell’s physiological requirements, 60 mg/g cells), total cell concentration increased. Specific growth rate of the cells calculated from the catalytic biomass during cultivation was 0.13 h^−1^. Specific rate of polymer synthesis (µ_p_) in the first stage of the culture was close to 0.03 h^−1^; by the end of the first stage it had increased to 0.1 h^−1^, reaching its maximum (0.14 h^−1^) by the middle of the second stage. Intracellular polymer content reached 45% and cell concentration (27±2) g/L.

In the second stage, cells were grown in nitrogen-free medium, with glucose supply regulated to maintain its concentration at 5 g/L. In this stage, specific growth rate (μ) decreased gradually, and in the last hours of cultivation it was 0.05 h^−1^; also, the rate of polymer synthesis had decreased to 0.04 h^−1^ by the end of the experiment. At the end of the experiment (50 h), intracellular polymer concentration reached 80–85% and the total cell concentration (42±2) g/L. The total amounts of glucose used to form catalytic cell biomass and polymer were (12.5±0.1) and (3.1±0.1) g/g, and their yield coefficients were 0.08 and 0.32 g/g, respectively. Productivity of cultivation was 0.17 g/L h of catalytic biomass and 0.67 g/L h of the polymer.

### The influence of physiological activity of C. eutrophus B-10646 inoculum on the lag- phase length, final cell concentration, and polymer yield

Relationships between physiological activity of the inoculum, which is determined by intracellular polymer concentration (10, 20, and 30%), and cellular respiration rate (20.0, 17.5, and 14.3 mg O_2_/g), on the one hand, and cell catalytic biomass accumulation dynamics and polymer yield, on the other, are shown in Figure 1B. Initial cell concentration in the inoculum was 3.0–3.5 g/L in all cases. Curves in Figure 1B show that final concentration of total biomass varied significantly, depending on parameters of the first stage of cultivation, which, in turn, depended on physiological activity of the inoculum. If initial polymer concentration in the inoculum did not exceed 10% (and cellular respiration rate was 20 mg O_2_/g h), after 2–4 h, cell concentration in the culture increased while glucose and nitrogen concentrations in the culture medium decreased. After 20–25 h of cultivation, at the end of the first stage, cell concentration reached 37 g/L, and intracellular polymer concentration was similar to that of the previous experiment (about 50%). At higher intracellular polymer content (20 and 30%), physiological activity of the inoculum was reduced to 17.5 and 14.3 mg O_2_/g h, respectively. Curves of cell biomass accumulation in Figure 1B look somewhat different from each other. At initial polymer concentrations in the inoculum 20 and 30%, the curves show latent phases lasting 8 and 12 h, respectively. Hence, cell concentrations at the end of the first stage were 17.5; 25.0; and 32 g/L, respectively. That did not cause a reduction in intracellular polymer content, which reached about 50% in all cases. In the second stage, no nitrogen was fed to the culture; glucose was added to the culture medium with a peristaltic metering pump, in amounts determined by cell concentration. The entire fermentation process (50 h) yielded cell concentrations varying from 38 to 62 g/L, depending on physiological activity of the inoculum, and similar polymer concentrations. Production parameters of the process ranged from 0.8 to 1.24 g/L h of the cells and from 0.64 to 0.99 g/L h of the polymer. Yield coefficients for glucose varied from 0.28 to 0.32 g/g. Thus, experiments in which the first stage of cultivation was conducted taking into account polymer content of the inoculum resulted in higher total cell biomass production.

### The influence of cell concentration in the inoculum on growth of C. eutrophus B-10646 culture and PHA synthesis

The first stage of the special two-stage cultivation mode used to synthesize PHAs produces maximum cell concentration under limited nitrogen supply. However, if cell concentration in the inoculum is too high and intracellular polymer content is increased during cultivation in a shake flask, the process in the fermentor is less efficient as the lag phase becomes longer and production parameters of the culture are decreased.

In order to maximize cell concentration in the first stage of the process, we studied the effect of the initial concentration of the inoculum on culture parameters. The inoculum was prepared as follows: the museum culture was grown for 15–20 h in 2-L flasks to obtain cell concentration that did not exceed (1.3±0.1) g/L and intracellular polymer content that was no higher than (8.0±0.5)%. Then, the cells were aseptically separated in an Avanti high-speed floor centrifuge and resuspended in mineral medium. The resulting inocula had different cell concentrations.

Relationships between cell concentration in the initial inoculum and cell biomass increase in the culture of *C. eutrophus* B-10646 cells synthesizing P3HB are shown in Figure 1C. Inocula used in the experiment had cell concentrations 2.5, 4.0, and 7.0 g/L. The amounts of glucose and nitrogen fed into the culture medium and intervals at which these substrates and solutions of major and minor mineral elements were added to the medium were regulated depending on cell concentration. Aeration of the culture medium was also automatically regulated, taking into account physiological requirements of the cells and the inflow of glucose. At the end of the first stage, cell concentrations reached 20.0, 37.0, and 53.0 g/L, respectively. In the second stage, cells were grown in nitrogen-free medium, under maximum aeration, with glucose and minor and major elements fed to the culture medium for 25 h; final cell concentrations reached 43.0, 62.0, and 107.5 g/L respectively. Different cell concentrations did not affect of intracellular polymer accumulation. In all cases, final P3HB concentration reached 80–85%. Variations in cell concentrations of the inoculum, under regulated aeration of the culture medium and regulated fluxes of glucose and mineral elements, significantly affected process productivity. With initial cell concentrations of the inocula 2.5, 4.0 and 7.0 g/L, process productivity reached 0.86, 1.24, and 2.15 g/L·h of cells and 0.69, 0.99, and 1.72 g/L·h of PHA, respectively. Glucose consumption averaged 2.5±0.1 g/g cells and 3.5±0.1 g/g polymer.

### A study of conditions for synthesis of PHA terpolymers

By varying the conditions of carbon nutrition, the process of cultivation of *C.eutrophus* B-10646 cells described above was adapted to produce PHA copolymers. Strain B-10646 shows enhanced tolerance to valerate, hexanoate, propionate, and γ-butyrolactone, and, thus, it was used to produce copolymers with different chemical structures in experiments with two precursor substrates – potassium propionate and γ-butyrolactone or potassium valerate and potassium hexanoate – simultaneously added to the culture medium.

Culture conditions and process parameters were the same as in the case of cultivation for P3HB synthesis. Carbon nutrition conditions were changed: in the early phase of the first stage (6–8 h after the beginning of cultivation in the fermentor), with nitrogen fed to the culture medium and cells cultured in complete nutrient medium, we added precursor substrates. Precursor substrates were added when intracellular polymer concentration was rather low, about 15–20%. They were added in 2–3 portions at intervals of one to three hours. Concentrations of each precursor substrate in the culture medium varied from 1–2 to 3–5 mg/L.

**Figure 2:**
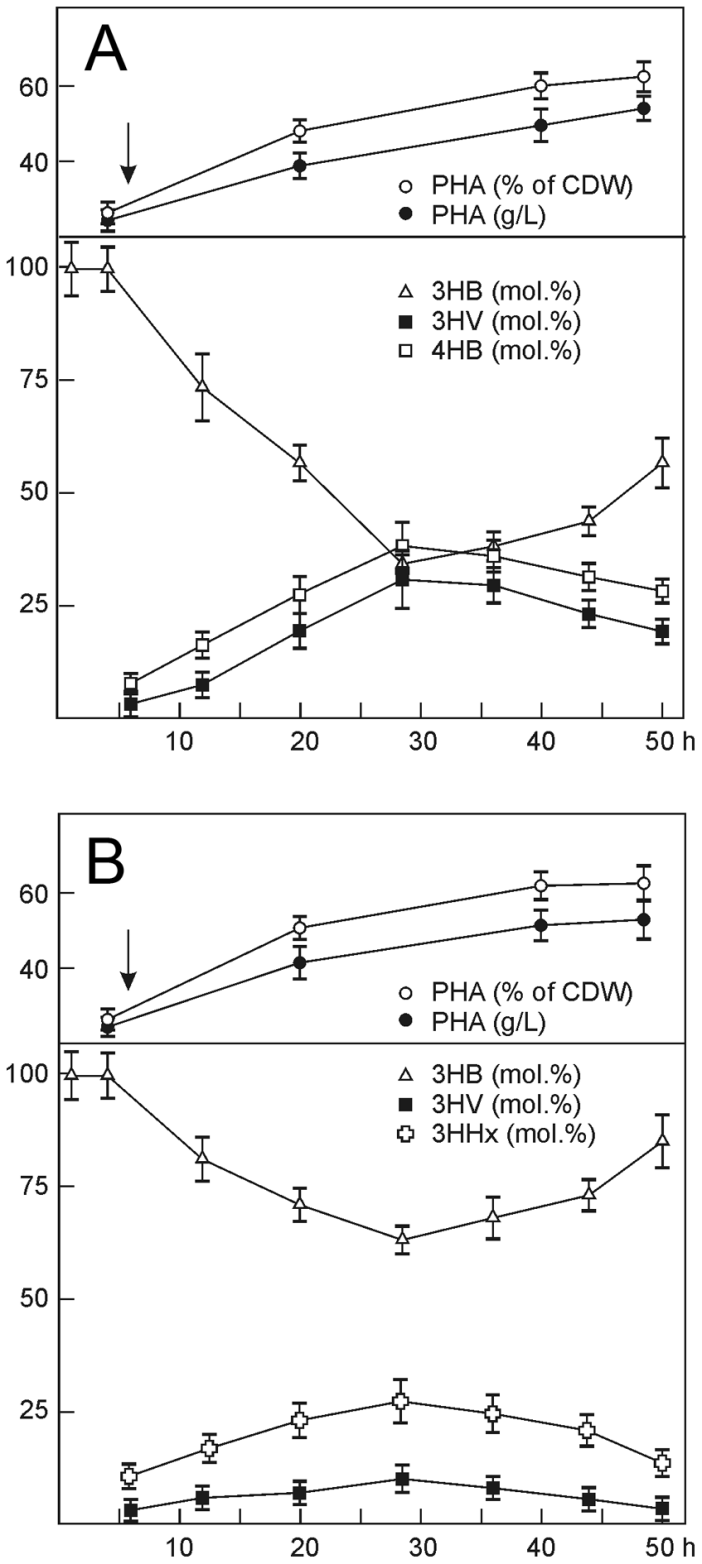
Parameters of the *C. eutrophus* B-10646 culture and dynamics of monomer fractions in the PHA in experiments with mixed carbon substrate: glucose+propionate+γ-butyrolactone (A) and glucose+valerate+hexanoate (B). (↓) The arrow shows additions of precursor substrates to the culture medium (6–8 h after the beginning of cultivation).

Changes in cell concentration, PHA accumulation, and PHA monomer fractions are shown in Figure 2. Cell biomass yield in the experiment with the addition of precursor substrates was lower than in the experiment with the cells grown on sole carbon substrate (glucose). As the composition of PHA heteropolymers is unstable, the highest molar fractions of such monomer units as 3HV, 4HB and 3HHx may be obtained only at certain time points after the addition of precursor substrates to the culture medium, as we reported elsewhere [17]. As shown in Figure 2, monomer fractions of PHAs changed during the course of cell cultivation, and the highest molar fractions of 3HV, 4HB, and 3HHx were achieved 20–25 h after the addition of precursor substrates to the culture medium; then they decreased while the molar fraction of 3HB increased. Therefore, in order to obtain PHAs with major fractions of certain monomer units, it is necessary to control not only the amounts of precursor substrates added to the culture medium but also the length of cultivation following the addition of these substrates.

Using the approach described above, we synthesized PHA terpolymers with different structures, which contained major molar fractions of 3HB (26.2 to 92.6%), 3HV (1.3 to 26.1%), 4HB (26.3 to 60.4%), and 3HHx (2.5 to 13.6%) (Table 1).

**Table 1:** Chemical composition and properties of PHAs synthesized by *Cupriavidus eutrophus* B-10646 from glucose supplemented with precursor substrates: propionate+γ-butyrolactone (Samples 1–5) or valerate+hexanoate (Samples 6–8).

Sample No.	PHA composition (mol. %)				M_n_ (kDa)	M_w_ (kDa)	<	T_m_ (°C)	T_d_ (°C)	C_x_ (%)
	3HB	3HV	4HB	3HHx						
P3HB	100	0	0	0	365	913	2.5	179	285	76
1	55.2	18.5	26.3	0	176	669	3.8	171	282	21
2	57.6	11.9	30.5	0	181	724	4.0	168	283	20
3	59.4	7.2	33.4	0	150	450	3.0	161	275	22
4	47.3	17.7	35.0	0	138	483	3.5	160	274	9
5	26.2	13.4	60.4	0	149	507	3.4	158	274	17
6	71.4	26.1	0	2.5	147	529	3.6	175	262	53
7	92.6	1.3	0	6.1	210	546	2.6	173	272	62
8	84.6	1.8	0	13.6	225	924	4.1	172	270	63

### Properties of PHA terpolymers with different chemical composition

**Figure 3:**
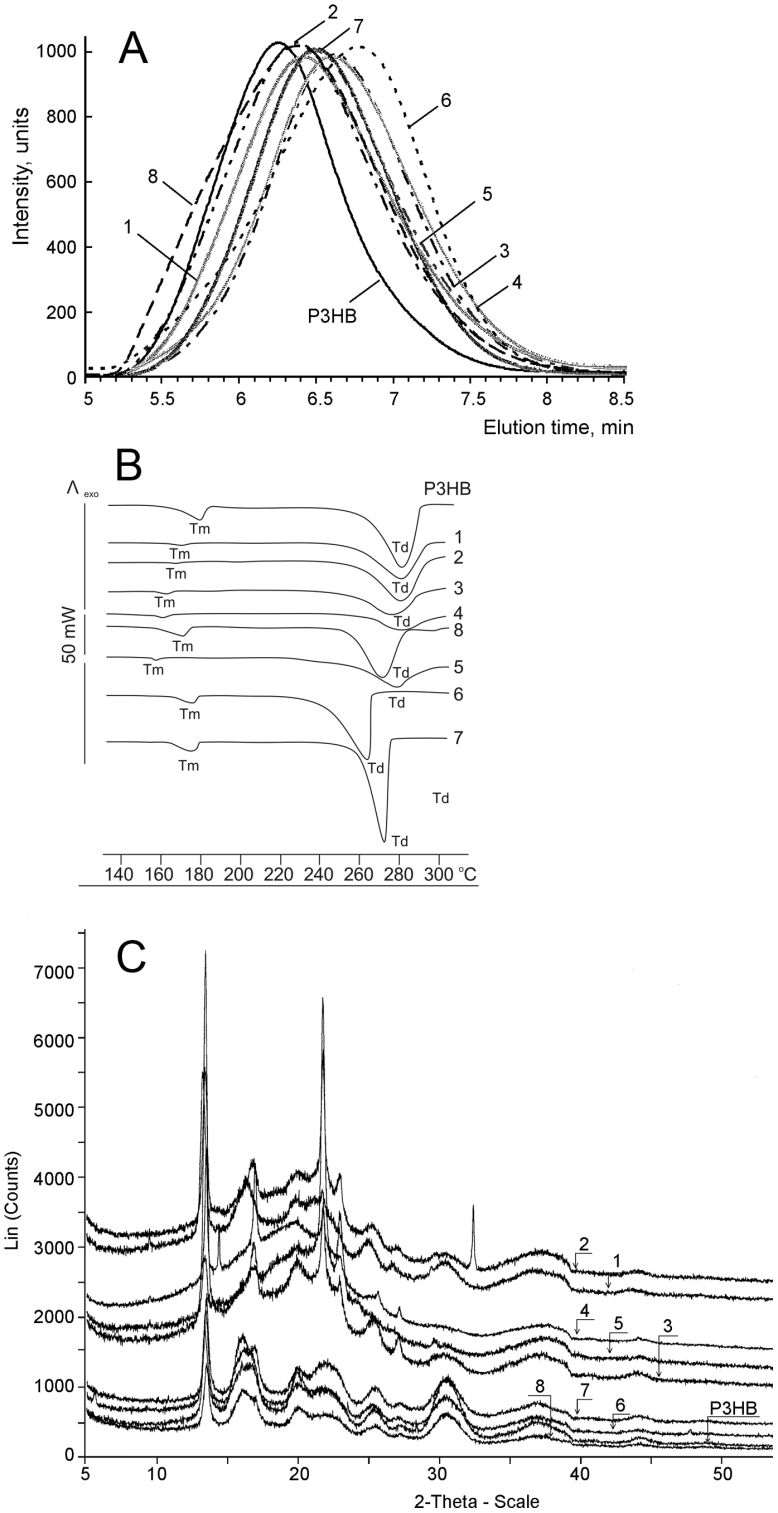
Physicochemical properties of PHA terpolymers: molecular weight characteristics (A), DSC (B), and X-Ray (C).

**Physicochemical properties:** Table 1 and Figure 3 compare physicochemical properties of different PHA terpolymers investigated using gel permeation chromatography, X-ray structure analysis, and differential scanning calorimetry.

It is well-known that PHA molecular weight is a very variable parameter, depending on a number of factors, including carbon source used, the length of cultivation, and the technique of polymer recovery employed [36], [37]. No clear relationship was found between PHA composition and values of M_w_ and M_n_. The M_w_ of the terpolymers ranged from 450 to 924 kDa and their M_n_ values – from 138 to 225 kDa (Table 1), but both parameters were lower in the terpolymers than in the P3HB homopolymer (913 and 365 kDa). Figure 3A shows chromatograms of molecular weight distribution of PHA terpolymers with different composition. PHA polydispersity values, which provided an estimate of the proportions of fragments with different polymerization abilities in the polymer, ranged from 2.6 to 4.1 and were higher than P3HB polydispersity (Table 1).

Temperatures of successive phase transitions for PHA terpolymers, determined by DSC, are given in Figure 3B, showing that for the majority of PHA terpolymers both T_m_ and T_d_ were lower than the corresponding temperatures of the P3HB homopolymer, whose melting temperature ranges from 160 to 185°C, with the T_m_ peak at 176–182°C, and thermal decomposition temperature range is between 260 and 290°C, with a narrow peak at 285 °C. The melting temperature of the P(3HB/3HV/4HB) copolymers was lower than the melting peak of the homogenous P3HB, and it became even lower as the fractions of 3HV and 4HB increased while the fraction of 3HB decreased. The peaks of melting and thermal decomposition temperatures of P(3HB/3HV/3HHx) copolymers were also somewhat lower than the corresponding peaks of P3HB. It is important to note that while melting temperatures and thermal decomposition temperatures of all PHAs studied decreased, the difference between them remained almost unchanged. Hence, the PHA terpolymers containing varying fractions of different monomer units retained one of the most important properties of PHAs – they remained thermoplastic.

The degree of crystallinity of polymers (C_x_) is most significantly influenced by the composition and monomer fractions of PHA terpolymers (Fig. 3C). The P(3HB/3HV/4HB) terpolymers showed the lowest degree of crystallinity, 9–22% (Table 1). The difference between the C_x_ of P(3HB/3HV/3HHx) copolymers and P3HB, a highly crystalline polymer, was less pronounced: 53–63% and 76%, respectively. In all PHA terpolymers, the crystalline phase decreased while the amorphous, disordered regions increased, indicating better processability of these polymers.

Thus, *C. eutrophus* B-10646 cells grown in standard two-stage batch culture on glucose, with precursor substrates added to the culture medium, synthesized PHA terpolymers that contained various monomer fractions and had different physicochemical properties.

**Figure 4:**
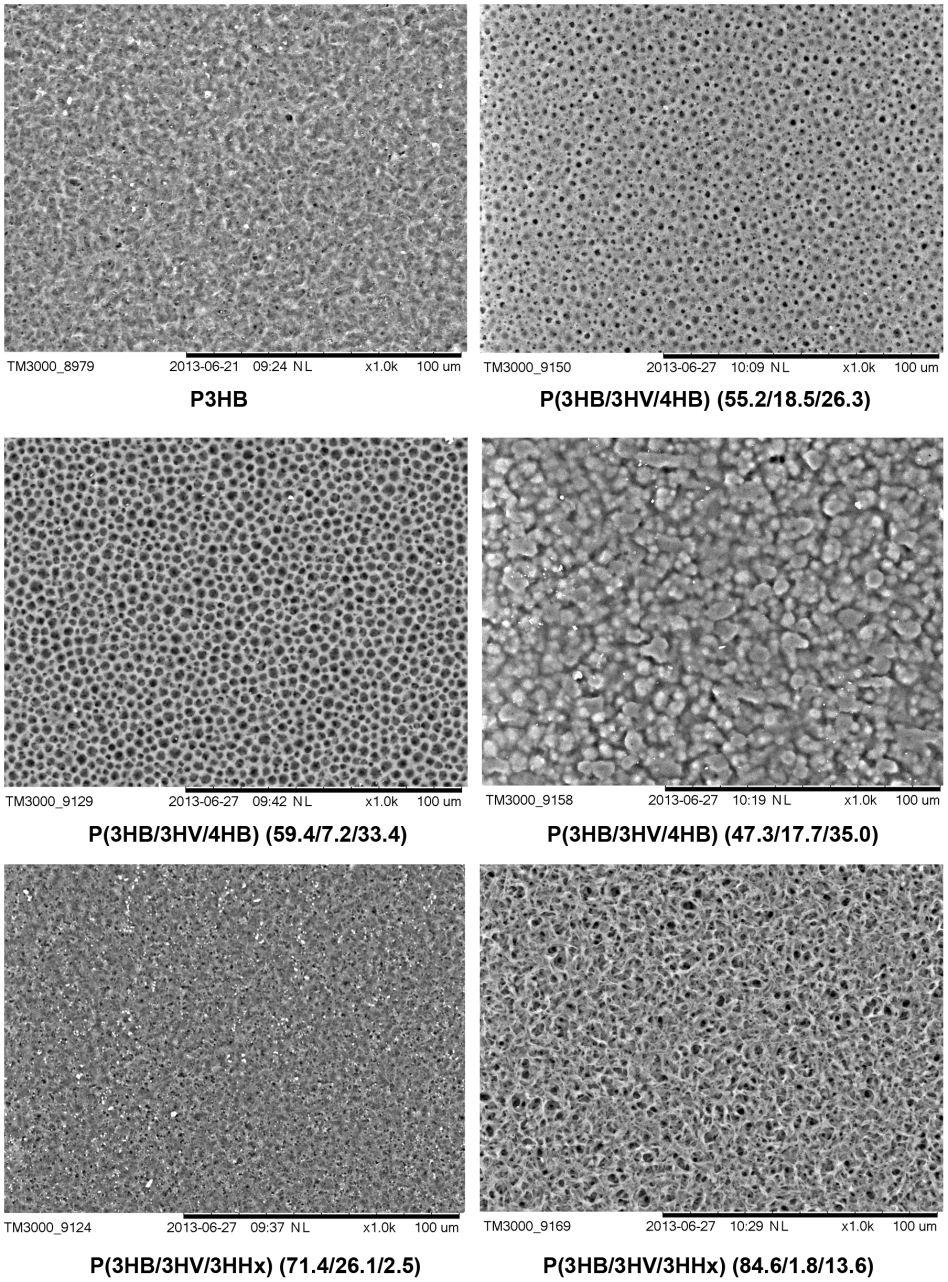
SEM images of surface topography of polymer films prepared from PHAs with different chemical composition. Bar  =  100 µm.

**Surface structure and properties of PHA films**: Differences in physical properties of the PHA terpolymers influenced the properties of the films prepared from these PHAs. Electron microscopy of the surface structure of films prepared from PHAs that differed in their chemical composition and basic physicochemical properties showed certain dissimilarities (Fig. 4). The surface of the films prepared from the P3HB homopolymer was nearly smooth with a few pores of diameter no more than 1 µm; on the surface of the films prepared from P(3HB/3HV/4HB) (55.2/18.5/26.3), there were numerous round pores of diameter about 1–4 µm. On the surface of the films prepared from P(3HB/3HV/4HB) (59.4/7.2/33.4), the pores were larger, reaching 5 µm, and more homogeneously sized. As the 4HB molar fraction increased, the film surface changed: the sample containing 3HV (17.7%) and 4HB (35.0%) had no pores, and the surface was covered by variously shaped protuberances and bubbles. The surface topography of the films prepared from the copolymer with 26.1 mol.% 3HV and 2.5 mol.% 3HHx was similar to the surface of P3HB films, but the surface of the P(3HB/3HV/3HHx) (84.6/1.8/13.6) sample had a rougher structure, with numerous pores reaching 4 µm.

Hydrophilic/hydrophobic balance of the surface is a major parameter that indirectly characterizes biological compatibility and influences cell adhesion and viability [38]–[39]. The highest value of contact angle (70°00′) was recorded for P3HB films (Table 2).Films prepared from PHA terpolymers had larger water contact angles. The water contact angles of the samples that contained not only 3HB and 3HV but also 3HHx [P(3HB/3HV/3HHx)] were the greatest (above 90°). The terpolymers of the other type [P(3HB/3HV/4HB)], which contained 3HV and 4HB, showed similar water contact angles – 79°10′-89°20′. These values were somewhat higher than the water contact angle of polystyrene culture plates (67°12′).

**Table 2:** Surface properties of films from solutions of PHAs with different chemical compositions.

Sample No.	PHA composition, mol. %	θ	γ (erg/cm^2^)	*W*_SL_ (erg/cm^2^)	γ_SL_ (erg/cm^2^)	R_a_ (nm)	R_q_ (nm)
–	P3HB (100)	70°00	32.78	97.70	7.88	71.75	80.28
1	P(3HB/3HV/4HB) (55.2/18.5/26.3)	79°10	25.73	86.56	11.97	113.74	153.39
3	P(3HB/3HV/4HB) (59.4/7.2/33.4)	84°06	22.16	80.33	14.63	43.32	56.24
4	P(3HB/3HV/4HB) (47.3/17.7/35.0)	89°20	18.71	73.81	17.7	172.37	206.06
5	P(3HB/3HV/4HB) (26.2/13.4/60.4)	82°92	22.96	81.77	13.99	63.88	74.69
6	P(3HB/3HV/3HHx) (71.4/26.1/2.5)	92°72	16.51	69.34	19.97	19.54	22.89
8	P(3HB/3HV/3HHx) (84.6/1.8/13.6)	96°82	14.13	64.15	22.78	208.29	243.62

Surface energy is another major parameter that can influence the behavior of cells [40]–[42]. However, the hydrophilic/hydrophobic balance of the surface, which determines surface energy and other parameters, does not exert a universal effect on cells: in some cases functions of cell structures are enhanced on hydrophilic surfaces, while in other cases – on hydrophobic ones [43]. All copolymer films had lower values of surface tension and cohesive forces than P3HB and higher values of interfacial free energy, 11.97 to 22.78 erg/cm^2^.

**Figure 5:**
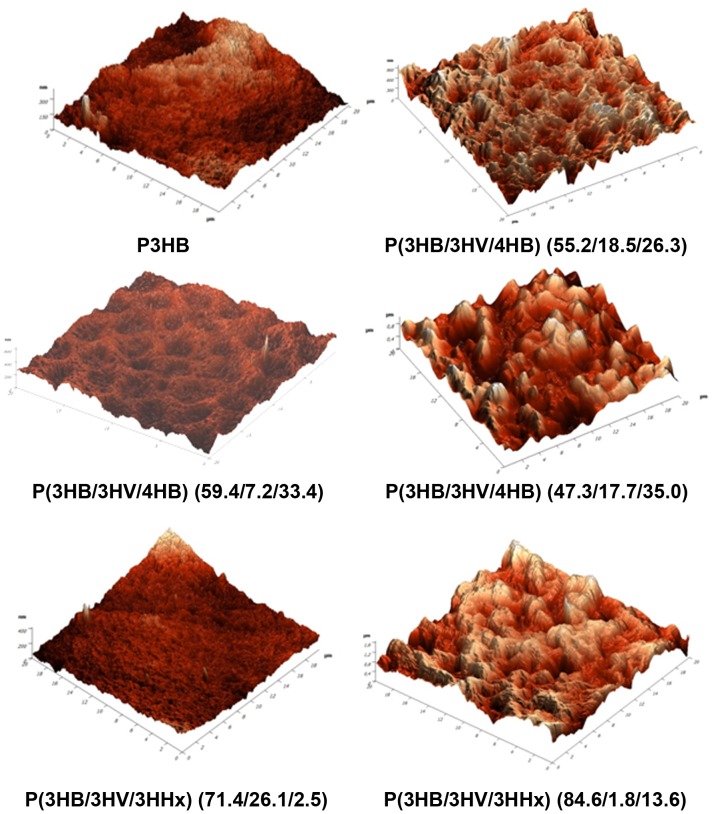
AFM of surface topography of polymer films prepared from PHAs with different chemical composition.

It is well-known that nanoscale surface roughness determines cell attachment, spreading, and motile activity and affects synthesis of specific proteins [44]. However, some data suggest that rough surfaces favor cell attachment more effectively than polished ones, while other data indicate that changes in the surface roughness do not cause any changes in cell behavior [45]. Results of investigating the surface roughness of the films prepared from PHA heteropolymers are given in Figure 5 and Table 2. The values of root mean squared roughness (Rq) of the films prepared from PHA terpolymers differed depending on the monomer units constituting the PHA and their molar fractions. The highest Rq values (243 nm) were recorded in the P(3HB/3HV/3HHx)  =  84.6/1.8/13.6 mol.% sample, and that was almost 3 times higher than the Rq value of the films prepared from the P3HB homopolymer (80 nm). The lowest Rq value was recorded for the sample containing different molar fractions of the same monomer units – P(3HB/3HV/3HHx)  =  71.4/26.1/2.5 mol.%. Samples consisting of 3HB (50–55 mol.%), 4HB (30–35 mol.%), and 3HV (17–18 mol.%) monomer units (Samples 1 and 4 in Table 2) had relatively high Rq values, 153 and 206 nm. At the same time, the roughness of PHA terpolymers containing different molar fractions of the same monomer units (Samples 3 and 6 in Table 2) was significantly lower, 56 and 22 nm, respectively. These differences in the roughness are clearly demonstrated by results of AFM (Fig. 5). These results differ from those obtained for PHA biopolymers: their Rq values varied from (82, 37 nm) as reported by Volova et al. [22] to (165 nm) as reported by Boskhomdzhiev [46].

**Physical-mechanical properties of PHA terpolymers:** Table 3 compares physical-mechanical properties of PHA terpolymers with different composition and monomer fractions. All copolymer films had significantly (1–2 orders of magnitude) higher values of elongation at break than films prepared from the highly crystalline poly-3-hydroxybutyrate. The presence on 3HHx monomer units in the polymer was the major factor influencing this parameter. Films with relatively low 3HHx molar fractions (2.5%) and relatively high 3HV fractions (26.1%) showed relative elongation at break of 73.2%, while the films with 13.6 mol.% 3HHx and low 3HV (1.8 mol.%) had much higher ε – 390.5%. A similar effect was observed for the 4HB fraction: as the molar fraction of 4HB was increased from 26.3 to 60.4%, elongation at break of the films increased from 231.5 to 371.1%. Higher elasticity of PHA terpolymers was accompanied by lower mechanical strength of the films, whose Young’s modulus was an order of magnitude lower than that of P3HB films. The tensile strength of the terpolymer films was, however, only 1.5–2 times lower than that of P3HB films.

**Table 3:** Physical-mechanical properties of films from solutions of PHAs with different chemical composition.

Sample No.	PHA composition (mol. %)	E (MPa)	σ (MPa)	ε (%)
–	P3HB (100)	2071.2	16.7	2.5
1	P(3HB/3HV/4HB) (55.2/18.5/26.3)	239.3	8.1	231.5
5	P(3HB/3HV/4HB) (26.2/13.4/60.4)	37.5	10.1	371.1
6	P(3HB/3HV/3HHx) (71.4/26.1/2.5)	312.3	10.8	73.2
8	P(3HB/3HV/3HHx) (84.6/1.8/13.6)	257.5	9.4	390.5

Thus, mechanical properties of polymer films can be altered by varying the composition and fractions of monomer units in PHAs.

**Figure 6:**
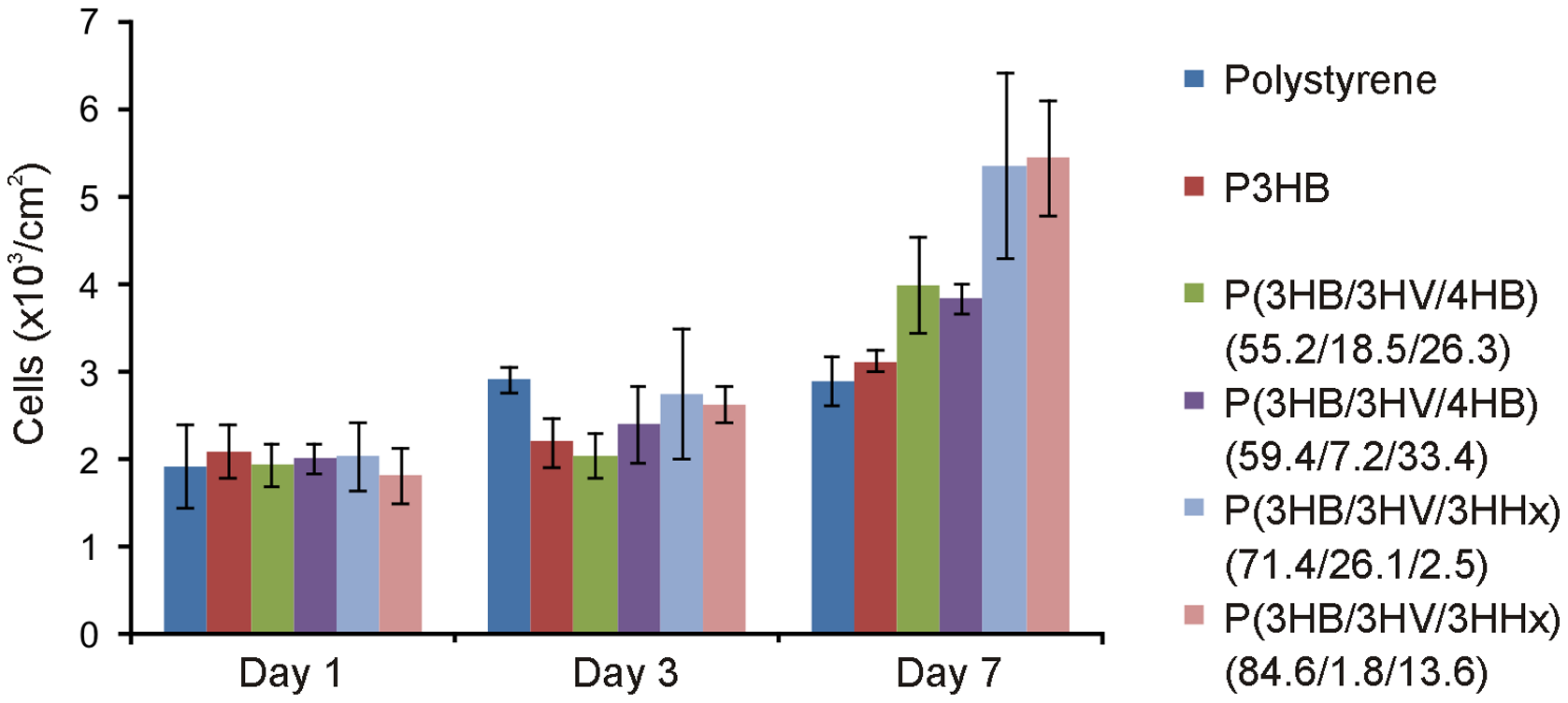
Results of MTT assay of mouse fibroblast NIH 3T3 cells cultured on films prepared from PHAs with different composition.

**Assays of cell attachment and viability on films from PHAs with different chemical compositions:** MTT assay did not reveal any cytotoxic effect of PHA films as compared to the reference polystyrene films (Fig. 6). More significant differences were observed at Day 7 of the experiment, when on all terpolymer films the number of cells was greater than on the reference film. The greatest number of viable cells was recorded on PHA films that contained not only 3-hydroxybutyrate and 3-hydroxyvalerate but also 3-hydroxyhexanoate (2.5 and 13.6 mol.%) – 5.36±1.06×10^3^ and 5.45±0.66×10^3^ cells/cm^2^, respectively; these counts were 1.8 times higher than on polystyrene and 1.7 times higher than on P3HB films.

**Figure 7:**
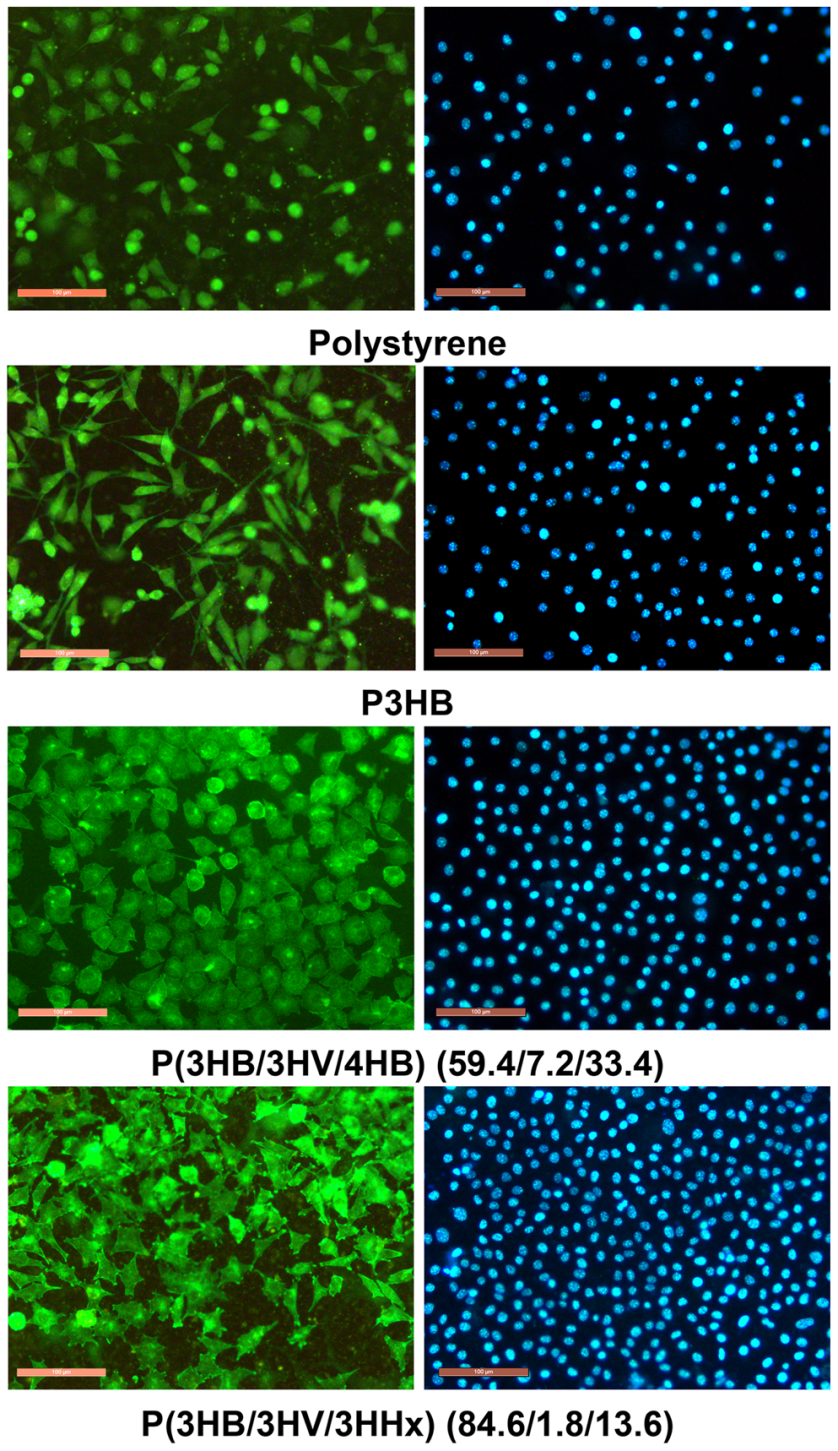
Morphology of mouse fibroblast NIH 3T3 cells cultured on films prepared from PHAs with different composition at Day 7 of the culture: FITC and DAPI staining. Bar  =  100 µ**m.**

Results of morphological investigations of fibroblast cells proliferating on films prepared from PHAs with different composition using the fluorescent DAPI DNA stain and FITC cytoplasm stain were generally similar to those obtained using MTT assay (Fig. 7). Thus, the best adhesion and proliferation of mouse fibroblast NIH 3T3 cells were obtained on films prepared from PHA terpolymers containing 3HHx; at the same time, all PHA films outperformed polystyrene and the P3HB homopolymer, which suggested their high biological compatibility with the cells cultured on them.

## Discussion

To achieve large-scale production of PHAs and extend their uses, it is necessary to develop effective processes of PHA biosynthesis, by employing highly productive strains and low-cost feedstocks. Among promising PHA producers are representatives of the genus *Cupriavidus*, which are capable of high-yield PHA synthesis on various substrates, including individual compounds and wastes [10].

The ability of *Cupriavidus* species to synthesize homogeneous poly-3-hydroxybutyrate (P3HB) from glucose was first reported in the late 1990s [47]. By now, industrial processes of P3HB synthesis from glucose with polymer yields reaching 80% and cell concentrations of 100–200 g/L have been developed and described [8], [11].

There is scant literature on the synthesis of PHA terpolymers by wild-type and genetically modified *Cupriavidus* strains, and the main carbon substrates used by the authors were oleic acid [48]–[50], glycerol [20], palm oil [26]–[27], and, more seldom, sugars [51], including fructose [4], [19] and glucose [52].

This study describes integrated investigations of growth kinetics of the new strain, *Cupriavidus eutrophus* B-10646, which shows enhanced tolerance to precursor substrates that are needed to synthesize 3HV, 4HB and 3HHx monomer units and is able to synthesize PHA copolymers from glucose. In two-stage batch culture, cells of this strain synthesized high yields of poly-3-hydroxybutyrate (P3HB), reaching 80-85%, from glucose. By optimizing certain parameters of the process (cell concentration in the inoculum; physiological activity of the inoculum, determined by initial intracellular polymer concentration), by controlling the glucose flux, and by pumping the air through the culture medium, we managed to obtain high yields of cells and polymer, above 110 g/L and 80±5%, respectively, in the process that lasted 45–50 h.

This study specifically focused on the ability of the strain to synthesize PHA terpolymers. Synthesis of PHA terpolymers by *Cupriavidus* species and by representatives of other taxonomic groups has been receiving more attention from researchers over the past few years, but the results reported in the literature were mainly obtained in flask cultures, which yielded low cell concentrations (1–2 to 6–8 g/L) and not very high PHA contents. PHAs were synthesized by the taxonomically close wild-type strains of *Cupriavidus* [50], *Wautersia eutropha* [53], and *Alcaligenes* [19], their mutant strains [20], and recombinant microorganisms [27], [54]–[57]. Bacteria were grown on fructose [19], [50], [53], oleic and palmitic acids, glucose, palm oil [50], and waste glycerol of biodiesel production [20]. Precursor substrates used in those studies were 1-propanol, 1-pentanol, valeric, nonanoic acids, γ-butyrolactone, 1, 4-butanediol, and sodium γ-hydroxybutyrate. In some cases, microorganisms were preliminarily cultured on rich media, containing peptone and meat and yeast extracts [19], [50], [53]. The most remarkable results of synthesis of PHA terpolymers that consisted of 3-hydroxybutyrate, 3-hydroxyvalerate and 4-hydroxybutyrate, were obtained in studies that used wild-type strains *Cupriavidus* sp. USMAA2-4 [50] and *Alcaligenes* sp. A-04a [19]. The authors of the former study obtained PHAs containing 23 mol.% 3HV and 26 mol.% 4HB by varying the composition of precursor substrates and the C/N ratio. PHAs obtained in the latter study contained even higher molar fractions of 3HV (up to 40%) and 4HB (50%). Madden et al. [52] investigated synthesis of P(3HB/3HV/4HB) in *Ralstonia eutropha* culture with high cell concentration grown on glucose in a fermentor; high final cell concentration (136 g/L) and PHA content reaching 62% were obtained, but molar fractions of 3HV and 4HB were low: 2 and 5%, respectively.

This study yielded better results. For instance, by adding carbon substrates to the culture medium portion-wise, taking into account the relationship between specific growth rates of *C. eutrophus* B-10646 cells and concentrations of precursor substrates revealed in this study, we managed to produce various PHA terpolymers, which contained total molar fractions of 3HV and 4HB exceeding 70%, with 3HV and 4HB fractions ranging from 7.2 to 18.5 and from 26.3 to 60.4 mol.%, respectively; cell concentrations reached 60–80 g/L and PHA yields 50 to 70%.

Synthesis of PHA terpolymers consisting of 3HB, 3HV, and 3HHx was a more difficult task. In a previous study, we cultivated *Ralstonia eutropha* B 5786 cells on fructose with potassium hexanoate and acrylate (which inhibits reactions of fatty acid β-oxidation cycle, thus preventing hexanoate disintegration and favoring its incorporation in the PHA) and obtained P(3HB/3HHx) copolymers containing over 50% 3HHx [17], [58]. In this study, however, when we added two precursor substrates (potassium valerate and potassium hexanoate) to the culture medium of *C. eutrophus* B-10646 cells synthesizing PHA on glucose, the resulting polymers contained 2.5 to 13.6 mol.% 3HHx and 1.3 to 26.1 mol.% 3HV (3HB being the major fraction).

Two groups of PHA terpolymers, containing different combinations and different molar fractions of monomer units, P(3HB/3HV/4HB) and P(3HB/3HV/3HHx), were used to prepare films, and then their physicochemical, physical-mechanical, and biological properties were investigated. As there are very few studies that simultaneously address PHA biosynthesis, production parameters of the culture, and composition and properties of polymers, the data on the properties of PHA terpolymers obtained in this study cannot be compared in detail with the literature data.

Using high-performance liquid chromatography, DSC, and X-ray structure analysis, we investigated physicochemical properties of PHA copolymers with different chemical composition. We did not reveal any significant influence of polymer composition on the molecular weight parameters of the PHAs, such as M_w_, M_n_, or polydispersity. This is in good agreement with the well-known opinion that these properties are rather influenced by the species composition of PHA producing strains, the age of the culture, and methods of polymer recovery and purification [9], [21]. The difference between the exothermal peaks was retained at a level of 80–100°C for all types of PHA copolymers, but their melting temperatures and thermal decomposition temperatures were lower than those of the P3HB homopolymer; moreover, as the 3HB molar fraction decreased and the fractions of other monomers increased, the melting points and thermal decomposition temperatures of the copolymers became even lower. The most significant differences were observed in the study of the degrees of crystallinity. The lowest C_x_ (9–22%) values were recorded for P(3HB/3HV/4HB) copolymers. Thus, in all PHA terpolymers, the crystalline phase decreased while the amorphous, disordered regions increased, indicating better processability of these polymers.

That was also confirmed by investigation of physical-mechanical properties of PHAs. The films prepared from PHA terpolymers all had higher mechanical strength and significantly (1-2 orders of magnitude) higher values of elongation at break than the films prepared from the highly crystalline P3HB. This study showed that P(3HB/3HV/4HB) terpolymers with higher molar fractions of 4HB had higher values of elongation at break but lower mechanical strength.

At the same time, a 1.5-fold decrease in the HV/HB fraction caused a considerable decrease in Young’s modulus – from 239.3 MPa to 37.5 MPa – and an increase in elongation at break from 231.5 to 371.1%. Ramachandran et al. [49] reported a similar change. The presence on 3HHx monomer units in the polymer was the major factor influencing this parameter. The relationship between P(3HB/3HV/3HHx) elasticity and molar fractions of 3HV and 3HHx revealed in this study is in good agreement with the data reported by Zhao et al. [54] and by Hu et al. [59]. In the latter paper, PHA terpolymers of the same composition as those investigated in this study had elongation at break 263.7%, Young’s modulus 284.6 MPa, and tensile strength 5.1 MPa, and these parameters were much higher than the corresponding characteristics of PHA bipolymers – P(3HB/3HV) and P(3HB/3HHx).

Differences in physical properties of the PHA terpolymers were reflected in the surface structure of polymer films prepared from them. The water contact angles of the terpolymer films varied from 79°10′ to 96°82′ and were greater than the water contact angle of P3HB films – 70°. A similar increase in both water contact angle and roughness of terpolymer films was shown in other studies [59], [60], which reported larger values of water contact angle (reaching 90–100°), interfacial free energy, and roughness of P(3HB/3HV/3HHx) terpolymers as compared with P(3HB/3HHx) and polylactide. In this study, however, investigation of film roughness did not reveal any clear relationship between the chemical composition of the PHA and Rq values.

The study of biological properties of PHA terpolymers was performed using mouse fibroblast NIH 3T3 cells, and neither MTT assay nor fluorescent DAPI and FITC staining revealed any cytotoxic effect of different PHA terpolymers, P(3HB/3HV/4HB) and P(3HB/3HV/3HHx), directly contacting with cells. Counts of physiologically active cells and examination of their morphology showed that the best results were obtained on the films prepared from PHAs that contained not only 3HB, but also 3HHx and 3HV. It is difficult to compare our results with the few available literature data because those data were obtained in different cultures: human bone marrow mesenchymal stem cells [59]–[62], keratinocytes [63]–[64], fibroblasts and osteoblasts [64]. Some of papers did not give exact data on the molar fractions of PHA monomer units [65] or PHAs used in them had different composition, with minor fractions of 3HHx, 3HV, and 3HB [59], [65]; those papers did not provide a characterization of the types and properties of the polymer carriers used (2D or 3D) or preparation techniques employed.

In spite of these limitations, we can conclude that results suggesting stronger attachment of cells to the films prepared from PHAs containing 3HHx and better cell proliferation on them are in good agreement with the available literature data. For instance, Wei et al. [59] showed that counts of human MSCs on P(3HB/3HV/3HHx); HaCaT keratinocytes on the P(3HB/3HV), P(3HB/3HHx), and P(3HB/4HB) [63]. Another type of PHA terpolymers, which contained 3HB, 4HB, and 3HHx, also facilitated more effective proliferation of MSCs. This type of PHA showed good results in the study by Liang et al. [65]: mouse fibroblast L929 cells, mouse osteoblast MC3T3-E1 cells, and human HaCat keratinocytes had better morphology and proliferated more actively on the terpolymer than on polylactide and P(3HB/3HHx).

Thus, PHA terpolymers synthesized and characterized in this study have enhanced biological properties for cell technologies, and these results are consistent with the literature data.

## Conclusion

This study investigated kinetic and production parameters of the culture of a glucose-utilizing strain, *C. eutrophus* B-10646. Optimization of bacterial culture, taking into account cell concentration and physiological activity in the inoculum, provided high cell concentrations and PHA yields under batch culture conditions and controlled carbon substrate supply. Addition of precursor substrates (valerate, hexanoate, propionate, γ-butyrolactone) to the culture medium enabled synthesis of PHA terpolymers with different composition and different molar fractions of 3HB, 3HV, 4HB,and 3HHx. PHA terpolymers had widely varying temperature parameters, degrees of crystallinity, and physical-mechanical properties; surface structure and properties of the films prepared from them also differed significantly. All films prepared from PHA terpolymers with different composition facilitated attachment and proliferation of mouse fibroblast NIH 3T3 cells more effectively than polystyrene and the highly crystalline P3HB.
